# Protection

**Published:** 1889-01

**Authors:** Lyman J. Gage


### PROTECTION.

### To the Members of the Dental Profession.

*Gentlemen:*—For the protection of our profession, and at the earnest solicitation of many of its prominet members, the undersigned undertook, after mature deliberation, the formation of "The Dental Protective Association of the United States." Its object at present is, in a lawful and equitable manner, to unite the strength of our profession to contest the patents of the International Tooth Crown Company, the validity of which has not been established.

Under competent legal advice we deemed it best to become incorporated. This has been accomplished under the name above set forth. The undersigned, having consented to act as the first Board of Directors, By Laws have been adopted, a copy of which is herewith enclosed to you. The number of Directors has been fixed at three, as it must be obvious to all that the work of such an organization can only be successfully handled by a small number not widely separated, so that there can be concerted action without delay.

Please examine some of the considerations which have led to the formation of this organization.

*First:* Dentists are writhing throughout the country under having to submit to a grasping monopoly because single handed and alone they cannot afford the expense of contesting its unjust claims. Numbers are being annoyed and prosecuted for the infringement of patents whose validity has never been legally established, and there is every reason to believe never can be established if dentists are organized for defense.

*Second:* Practitioners have not forgotten the treatment they received at the hands of the Goodyear Dental Vulcanite Company, and from the facts that the International Tooth Crown Company is largely managed by the same individuals, they can infer the treatment they may expect if they are unfortunate enough to be left in its power.

*Third:* If they do not defend themselves, but allow this company to prosecute its claims they will have to pay a royalty on all banded or gold crown they have ever made or may make in the future.

DON'T FORGET; that to yield to the company or to contest its demands alone and unaided, will cost you hundreds of dollars; that to protect yourself by joining the "Dental Protective Association" will probably cost you less than ten.

DON'T FORGET, that it will be impossible to reach every practitioner by circular, but notices will be sent to the Dental Journals and such notices are meant for you individually.

DON'T FORGET; that we want your name and membership fee of $10, and your hearty co-operation in securing your neighbors and friends, as members of this organization; that while the benefit of the organization will be shared by the many the work must in the main devolve upon a few. You can greatly aid the directors by responding promptly and by inducing other dentists to join the association.

We want five thousand names.

DON'T FORGET.—That name and address, *plainly written*, with membership fee, should be forwarded immediately to J. N. Crouse, Chairman of Board of Directors of the Dental Protective Association, 2231 Prairie Avenue.

Receipt will be forwarded at once, and a copy of by laws sent for you to sign, if you have not already done so.

Mr. Lyman J. Gage, Vice-President of the First National Bank of Chicago, Ill., has kindly consented to act as Treasurer for the Association.

| | |
|---|---|
| BOARD OF DIRECTORS. | J. N. CROUSE,<br>TRUMAN W. BROPHY,<br>E. D. SWAIN. |

LYMAN J. GAGE, Treasurer.

